# Bifunctional catalysis

**DOI:** 10.3762/bjoc.12.102

**Published:** 2016-05-25

**Authors:** Darren J Dixon

**Affiliations:** 1 Chemistry Research Laboratory, University of Oxford, Mansfield Road, Oxford OX1 3TA, UK, Tel: +44 (0)1865 275648; Fax: +44 (0)1865 285002

**Keywords:** bifunctional catalysis

Bifunctional catalysis concerns the use of low molecular weight, structurally defined molecules possessing two distinct functional groups to bring about new reactivity and/or selectivity in a reaction of interest. The reactions are typically polar addition reactions of pronucleophiles and electrophiles where, ideally, simple low-cost starting materials are converted into high-value, stereochemically defined products through the action of the bifunctional catalyst system.

Many bifunctional catalysts possess either Lewis or Brønsted basic functionality and a hydrogen-bond donor group suitably positioned over a chiral scaffold. Compared to single functional group catalysts, the cooperative effect of the two complementary functional groups can lead to new reactivity and stereocontrol in reactions that were previously challenging or unprecedented. With multiple points of diversity in the two functional groups and the chiral scaffold, these catalysts can be readily tuned to optimise reactivity and selectivity in synthetically relevant reactions. Furthermore, owing to the great number of pronucleophiles and electrophiles that are available, the number of polar addition reactions that are amenable to catalysis through the action of bifunctional catalysts is enormous, and consequently, the field continues to expand at an impressive pace.

The present Thematic Series serves to highlight the current state-of-the-art of bifunctional catalysis from new bifunctional catalyst design and development, their application in new asymmetric metholodgy development, to their application in natural product and drug target synthesis. The creativity and productivity of the researchers in the field in general, and the breadth of highly stereoselective reactions reported in this series in particular, is truly impressive, and I would like to express my sincere gratitude to all of the many contributors.

Darren J. Dixon

Oxford, April 2016

